# The *in Vivo* Antioxidant Effects of (−)-Epigallocatechin-3-Gallate Consumption in Healthy Postmenopausal Women Measured by Urinary Excretion of Secondary Lipid Peroxidation Products

**DOI:** 10.4236/fns.2019.101002

**Published:** 2019-01-04

**Authors:** Chelsey Fiecke, Mindy Kurzer, Chi Chen, A. Saari Csallany

**Affiliations:** Department of Food Science and Nutrition, University of Minnesota, St. Paul, MN, USA

**Keywords:** Antioxidants, Epigallocatechin-3-Gallate, Green Tea, Humans, Lipid Peroxidation, Urinary Excretion

## Abstract

The present study was carried out to determine whether the consumption of epigallocatechin (EGCG), the major bioactive green tea catechin, exerts a positive effect on lowering *in vivo* lipid peroxidation, a measure of oxidative stress, in healthy postmenopausal women. Urinary excretion of secondary lipid peroxidation products, a measure of *in vivo* lipid peroxidation, was determined in 40 participants randomly assigned to consume a green tea catechin extract (843.0 ± 44.0 mg EGCG/d) or placebo capsules for 12 months. Urine samples were analyzed for individual polar and nonpolar lipophilic aldehydes and related carbonyl compounds by high-performance liquid chromatography (HPLC) at the beginning and at the end of the 12-month intervention period. Results show that two nonpolar aldehydes, nonanal and decatrienal, were both 48% lower (p < 0.005) following consumption of EGCG. These results indicate that a modest degree of *in vivo* antioxidant activity exists with long-term EGCG consumption, which could slightly limit oxidative damage associated with lipid peroxidation and the onset and progression of chronic diseases.

## 1. Introduction

Green tea (GT) is a widely consumed beverage that has been associated with lower incidence of various chronic diseases, including cardiovascular and neurodegenerative diseases and certain types of cancer [1] [2] [3] [4] [5]. Since the prevalence of chronic diseases is a growing health concern affecting about 50% of adults in the United States [6], green tea could possibly aid in preventing or delaying the onset of these conditions [7]. The benefits of green tea in reducing chronic disease risk are supported by intervention studies in humans and chronic disease models in rodents [8]-[13]. In aging 14-month-old mice, 0.05% green tea catechins (71% epigallocatechin gallate [EGCG]) were administered in the drinking water for 6 months, which is equivalent to about 250 mg EGCG/day or 2 cups of GT/day for humans. In this mouse model, EGCG supplementation resulted in reduced levels of several oxidative stress markers in the hippocampus, including thiobarbituric acid reactive substances, a measure of aldehydic degradation products from lipid peroxidation, and protein carbonyls, superoxide dismutase, and glutathione peroxidase [9]. Furthermore, a short-term, 8-week human intervention study demonstrated attenuation of metabolic syndrome characteristics. Metabolic syndrome patients who consumed 4 cups GT/day had improvements in body weight, body mass index (BMI), low-density lipoprotein (LDL), and high-density lipoprotein (HDL). Body weight and BMI were significantly lower, while LDL tended to be reduced in patients consuming GT. Likewise, GT also caused a tendency towards increased HDL levels [8]. These studies indicate the beneficial effects of GT and, therefore, certain catechins such as EGCG.

The putative mechanism for the health benefits of GT seems to be the antioxidant function of its flavonoid constituents. Catechins represent 80% - 90% of the flavonoids in GT and their structures contribute to the antioxidant activity [14] [15]. The major catechins and their relative abundances are as follows: (−)-epigallocatechin-3-gallate (EGCG, 59%), (−)-epigallocatechin (EGC, 19%), (−)-epicatechin-3-gallate (ECG, 14%), and (−)-epicatechin (EC, 6%) [14]. Based on *in vitro* assays, EGCG has the most potent free radical scavenging activity of the catechins due to the presence of the phenolic rings and the trihydroxyl structure [16]. The antioxidant functions associated with EGCG have been demonstrated both *in vitro* and *in vivo* [8] [17] [18] [19] [20]. One recent *in vitro* study treated myocytes with 25 μM docosahexaenoic acid (DHA) to induce reactive oxygen species (ROS) production and mitochondrial fragmentation. Both of these DHA-dependent oxidative stress indicators were prevented by co-treatment of the myocytes with 25 μM EGCG [17]. Short-term human interventions in healthy subjects or patients with chronic diseases also support the antioxidant role of green tea [8] [19] [21]. Sung *et al.* [21] measured the total antioxidant capacity (TAC) in the plasma of healthy subjects using the ABTS antioxidant capacity assay. They demonstrated that consuming 450 mL of green tea increases plasma TAC by ~15% within 1 - 2 hours. Another short-term intervention study showed that consumption of green tea catechins (GTCs) caused a reduction in lipid peroxidation, an indirect measure of overall oxidative stress [8] [22]. In the study, patients with metabolic syndrome who consumed 900 mg catechins/day as a GT beverage demonstrated a reduction in serum hydroxynonenal (HNE) and malondialdehyde (MDA) concentration [8].

The present study was a sub-study of the Minnesota Green Tea Trial (MGTT), which was a double-blind, placebo-controlled, randomized clinical trial (clinicaltrials.gov; NCT00917735) [12]. The primary objective of the present study was to investigate changes in whole-body oxidative stress in response to long-term EGCG supplementation. This was accomplished by measuring urinary excretion of secondary lipid peroxidation products in healthy postmenopausal women following 12-month consumption of green tea extract (GTE) capsules containing ~800 mg EGCG. Measuring the level of whole-body oxidative stress is of interest because oxidative stress is related to various age-related chronic diseases, including cardiovascular and neurodegenerative diseases, and cancer [23] [24] [25] [26]. It was hypothesized that GTE supplementation would reduce *in vivo*lipid peroxidation and, therefore, the urinary excretion of lipophilic secondary oxidation products, such as aldehydes and related carbonyl compounds.

## 2. Materials and Methods

### 2.1. Materials

2,4-dinitrophenylhydrazine (DNPH), high performance liquid chromatography (HPLC)-grade acetone, dichloromethane, methanol (MeOH), and water were purchased from Sigma-Aldrich (St. Louis, MO). HPLC-grade hexane, acetone, and ACS-grade hydrochloric acid (HCl) were purchased from Fisher Scientific (Fair Lawn, NJ). Silica gel 60 thin layer chromatography plates (aluminum sheets, 20 × 20 cm, 200 μm) were purchased from EMD Millipore (Billerica, MA). Nylon membrane syringe filters (0.45 μm) were purchased from Whatman Ltd. (Maidstone, Kent, England). LC-MS-grade water and acetonitrile (ACN) were purchased from Thermo Fisher Scientific (Houston, TX) and ammonium acetate from Honeywell Riedel-de Haen (Seelze, Germany).

### 2.2. Subjects

Forty healthy postmenopausal women 50 - 70 years of age were randomly selected from the randomized, double-blind, placebo-controlled Minnesota Green Tea Trial (MGTT), which has been previously described [27]. Briefly, subjects for the MGTT were recruited from the Minneapolis-St. Paul area during August 2009-April 2013. Participants were randomized to consume either two decaffeinated GTE capsules or two placebo capsules, twice daily for 12 months. A total of 1075 women were randomized, with 454 in the placebo group and 424 in the GTE group completing the study. The average weight of the women at baseline was 67.4 kg for the GTE group and 67.9 kg for the placebo group. The average energy intake was 1438.3 kcal/d for the GTE group and 1447.5 kcal/d for the placebo group [27]. However, this estimate of energy intake is likely a significant underestimate, based on the metabolic needs of women of this body weight and age.

### 2.3. Experimental Procedures

Subjects consumed a self-selected diet *ad libitum* and either GTE or placebo capsules. Placebo and GTE capsules were supplied by Corban Laboratories (Eniva Nutraceutics, Plymouth, MN). Compliance was determined using the number of unused capsules and urinary levels of EGC and EC measured in 10% of the women at 0, 3, 6, 9, and 12 months [27]. The contents of the GTE and placebo capsules are shown in Table 1 and Table 2, respectively. Subjects randomized to the GTE group consumed 843.0 ± 44.0 mg EGCG/d, which represented 64% of the total catechin intake. This amount is equivalent to drinking about five 8-ounce cups of GT per day [12].

### 2.4. Urine Analysis of Excreted Secondary Lipid Peroxidation Products

For the present study, 24-hr urine samples were randomly selected from forty of the women who had completed the MGTT, twenty each from the GTE and placebo groups. The urine samples were frozen and stored at −20°C until analysis. Analysis of urinary excretion of nonpolar (NPCs) and polar (PCs) aldehydes and related carbonyls was accomplished using the method of Kim, Gallaher, and Csallany with minor modifications [28]. Briefly, the urine aliquots (3 - 4.75 mL each) were reacted with 6 mL DNPH reagent overnight at room temperature in a slowly oscillating shaker. The polar and nonpolar DNPH derivatives were extracted with dichloromethane followed by separation into NPCs, PCs, and osazone fractions by thin layer chromatography (TLC) on silica gel plates developed in dichloromethane. The nonpolar and polar DNPH derivatives were eluted from the TLC plates with 10 mL methanol three times. The pooled extracts were evaporated under N_2_ gas and concentrated to exactly 1 mL with methanol. Separation and quantification of the individual NPC and PC hydrazones was accomplished by HPLC using a mobile phase of MeOH (75):H_2_O (25) for NPCs and MeOH (50):H_2_O (50) for PCs. Isocratic elution proceeded for 10 min followed by a linear gradient to 100% MeOH for 20 min, followed by 100% MeOH for 10 min at a flow rate of 0.8 mL/min. Absorbance of NPCs, PCs, and related compounds was monitored at 378 nm. The HPLC system consisted of Varian 9010 solvent metering pump and sample injector and Varian 9050 UV detector. Separations were performed on an Ultrasphere ODS C18 reverse-phase column (25 cm × 4.6 mm i.d., 5 μm particle size) (Hichrom, United Kingdom) with a guard column (2.5 mm × 4.66 mm i.d.) (Grace & Co., Bannockburn, IL).

The total of the individual peak areas for the DNPH derivatives of NPCs and PCs was compared between the placebo and GTE groups at 12 months and between the GTE group at 12 months and both groups at 0 months. The individual NPC and PC peak areas were compared in the GTE group between 0 and 12 months. The individual NPC peaks that were significantly different in the GTE group between 0 and 12 months were isolated by collecting serial fractions from HPLC separations of pooled DNPH derivative samples that were concentrated to ~500 μL under N_2_ gas. The relative purity of the collected fractions was determined by comparing the number of peaks present in each fraction. Finally, the purified HPLC fractions were concentrated to ~500 μL under N_2_ gas for further analysis with ultra-performance liquid chromatography (UPLC) and mass spectrometry (MS).

### 2.5. Identification of the Individual DNPH Derivatives of Lipid Peroxidation Products

The DNPH derivatives of urinary metabolites were identified by LC-MS analysis. Briefly, samples were mixed with 5 volumes of 50% aqueous acetonitrile (ACN), and then transferred to HPLC vials. Five microliters of samples or solvent blank were injected into an Acquity ultra-performance liquid chromatography (UPLC) system (Waters: Milford, MA) and separated by a bridged ethyl-siloxane/silica hybrid (BEH) C18 column (Waters). Mobile phase gradients were A: H_2_O containing 0.1% formic acid (v/v), 10 mM ammonium acetate (pH 9) and B: ACN (95): H_2_O (5) containing 0.1% formic acid (v/v), 10 mM ammonium acetate (pH 9). The UPLC eluent was introduced into a Xevo-G2-S quadrupole time of flight mass spectrometry (QTOFMS) system (Waters) for ion detection and accurate mass measurement. The UPLC chromatograms and mass spectra were acquired and analyzed by MassLynx^™^ software (Waters) in centroided format. The structures of DNPH derivatives were determined by tandem (MS/MS) fragmentation with collision energy ranging from (10 - 50 eV) and elemental composition analysis.

### 2.6. Statistical Analysis

Statistical analyses were performed using R 3.4.1 (R Development Core Team, 2016). Paired *t*-tests were used for comparisons in each group between 0 months and 12 months. Welch’s two-sample *t*-tests were used for all other comparisons. The results are expressed as mean ± standard error of the mean (SEM). Results were considered statistically significant at *p* < 0.05.

## 3. Results

### Urinary Excretion of Nonpolar and Polar Secondary Lipid Peroxidation Products

**Figure 1.**
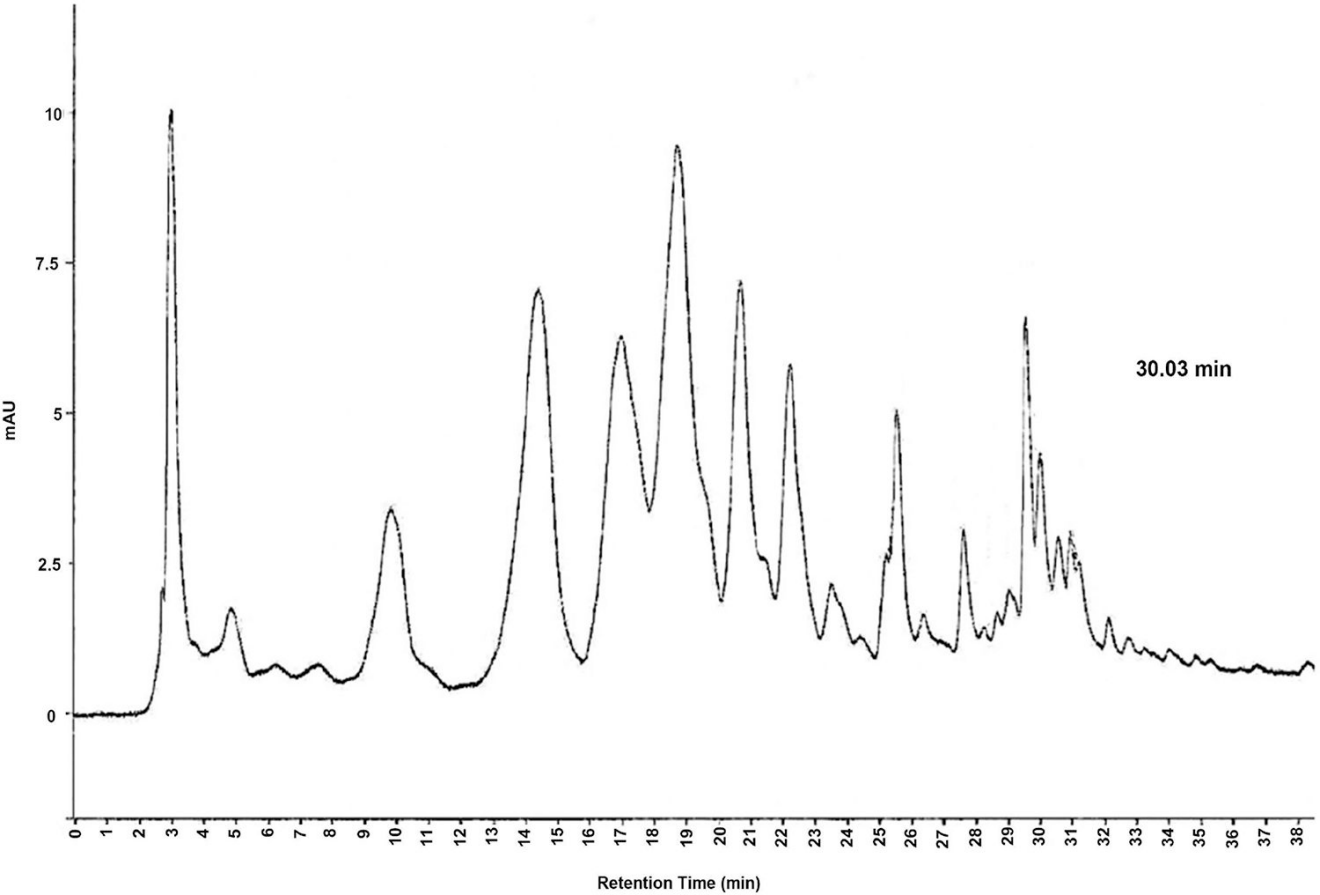
HPLC separation of the major DNPH derivatives of urinary nonpolar compounds (NPCs). Peak with retention time of 30.03 min has been shown to be significantly different following green tea extract (GTE) consumption for 12 months (p < 0.05). This peak was 48% lower in the GTE group at 12 months compared to both groups at 0 months.

**Figure 2.**
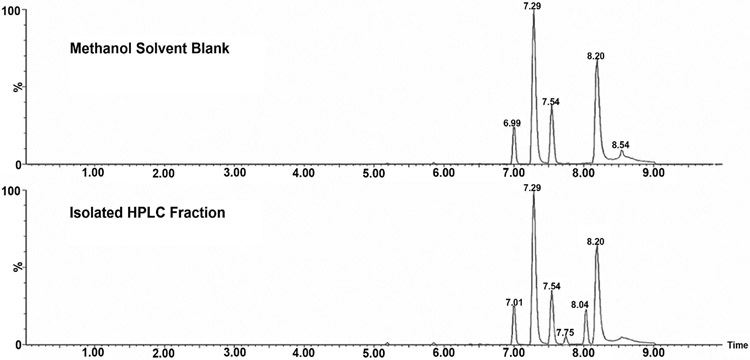
Ultra Performance Liquid Chromatography (UPLC) chromatogram for isolated HPLC fraction of peak eluted at 30.03 min (see Figure 1), demonstrating the presence of one minor compound with retention time 7.75 min and one major compound with retention time 8.04 min.

**Figure 3.**
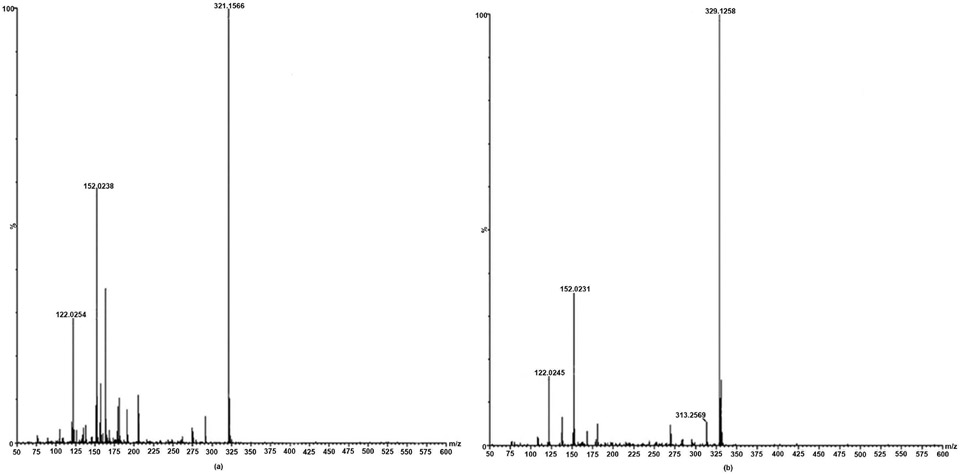
Quadrupole time-of-flight mass spectrometry (QTOFMS) for isolated HPLC fraction of peak eluted at 30.03 min (see Figure 1), identifying the major compound in the UPLC separation (see Figure 2) with retention time 8.04 min as (a) nonanal and the minor compound with retention time 7.75 min as (b) decatrienal.

**Figure 4.**
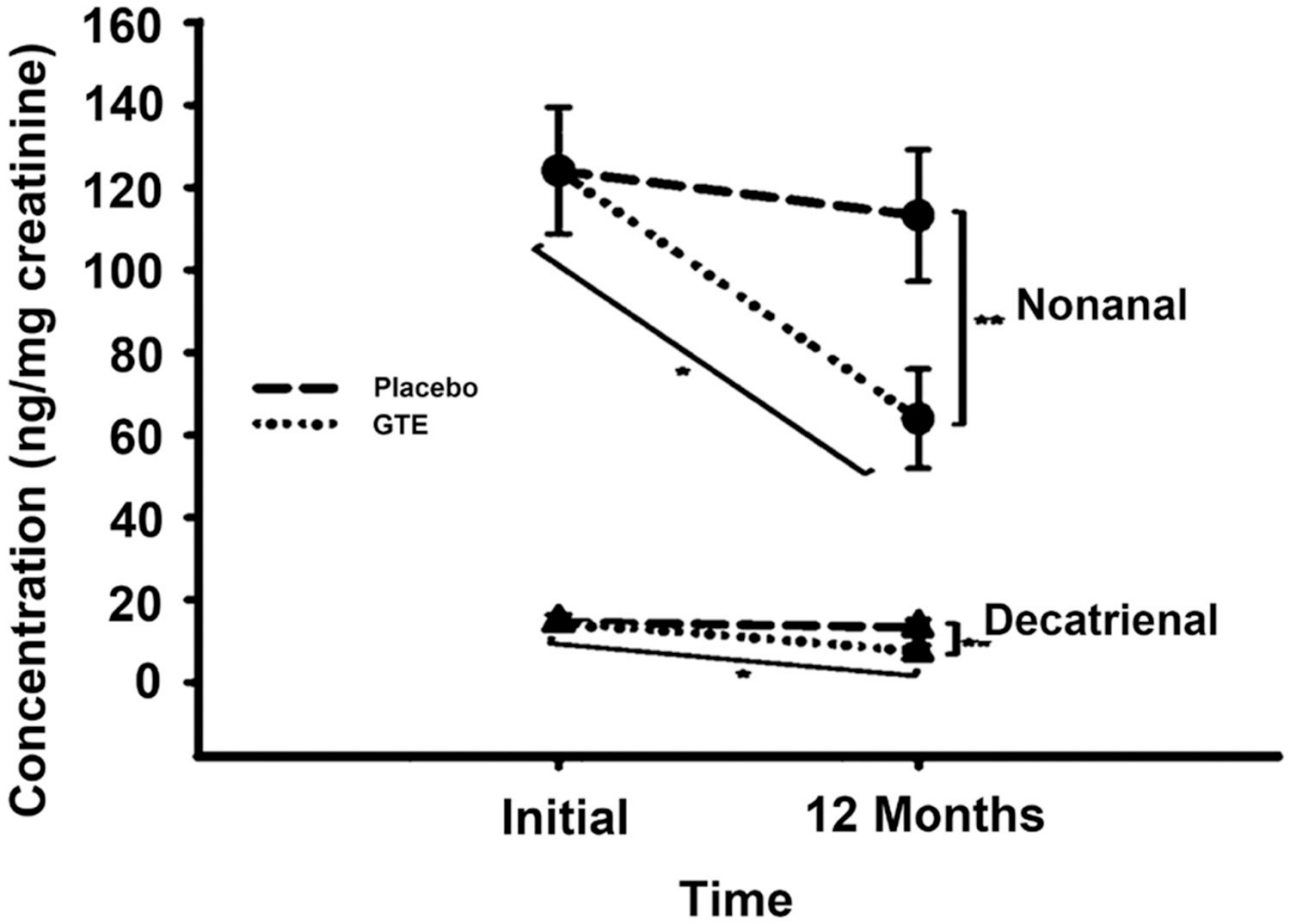
Nonanal and decatrienal concentrations before and after consumption of green tea extract (GTE) and placebo capsules. Values are means ± SEM for all subjects in each group (n = 20). *p < 0.005, when compared to both groups at 0 months. **p < 0.05, when compared to placebo group at 12 months.

Although the total of individually measured NPC peaks did not show significant differences after GTE consumption, comparison of the individual NPC peaks revealed a significant reduction for one peak after GTE consumption (Figure 1). This peak had a retention time of 30.03 min and was 48% lower in women consuming the GTE for 12 months compared to both groups at 0 months (p < 0.005). This peak was isolated and purified using HPLC prior to mass spectrometry analysis. Further purification was completed by UPLC, demonstrating the presence of one major peak with a retention time of 8.04 min and one minor peak with a retention time of 7.75 min (Figure 2). The molecular weight of these two compounds was determined by LC-MS (Figure 3), demonstrating that the major compound was nonanal (a 9-carbon saturated aldehyde), which represented 90% of the original HPLC fraction. The minor compound represented 10% of the original HPLC fraction and was identified as decatrienal (a 10-carbon tri-unsaturated aldehyde). These two nonpolar aldehydes were significantly lower following GTE supplementation for 12 months when compared to both groups at 0 months (p < 0.005) and the placebo group at 12 months (p < 0.05), as shown in Table 3 and Figure 4. The HPLC analysis of urinary PCs revealed no significant differences in any of the individually measured peaks between 0 and 12 months of GTE consumption.

## 4. Discussion

The objective of this study was to determine whether healthy, postmenopausal women consuming EGCG for 12 months would demonstrate a reduction of whole-body oxidative stress. The toxic secondary lipid peroxidation products that are formed *in vivo* and excreted in the urine demonstrate the extent of oxidative damage occurring in the body [29]. Our findings show that long-term supplementation of 800 mg EGCG/d produces a very slight reduction in lipid peroxidation *in vivo*. This is evidenced by the lower urinary excretion of nonanal and decatrienal, two aldehydic secondary lipid oxidation products. Similar reductions in the urinary excretion of individual NPCs has been shown in response to the consumption of other antioxidant dietary components, including the soy isoflavones genistein, daidzein, and glycitein [29]. The antioxidant effect shown in the present study in healthy postmenopausal women is also consistent with other short-term interventions involving GT or EGCG in healthy individuals and subjects with chronic disease [8] [19] [30]. Erba *et al.* showed that healthy women who consumed two cups of GT/d for six weeks had a significant 10% increase in plasma antioxidant activity that was inversely related to plasma peroxide levels, which were reduced by 25%. The study also demonstrated that GT consumption protected lymphocytes from oxidative damage to both lipids and DNA [30]. Likewise, Basu *et al.* showed that metabolic syndrome patients consuming a GT beverage for eight weeks had a statistically significant 0.39 μM reduction in serum levels of MDA and HNE [8].

*In vitro* studies indicate that the antioxidant effects of GTCs are directly related to the presence of the galloyl moiety and hydroxyl groups [15]. The trihydroxyl structure in EGCG contributes to its higher antioxidant activity and confers the capacity to scavenge lipid alkoxyl and peroxyl radicals [15] [31]. This allows GTCs to prevent oxidative damage to lipid membranes by limiting the propagation step of lipid peroxidation [31]. While various studies have confirmed the antioxidant potential of GTCs interventions in humans, most of these were only short-term investigations or conducted in patients with chronic diseases [8] [19] [32]. However, the present study is one of the first long-term studies to demonstrate an *in vivo* antioxidant potential for EGCG supplementation in a healthy population consuming a self-selected *ad libitum* diet, albeit a modest one.

Although EGCG supplementation resulted in less urinary excretion of nonanal and decatrienal, the expected reduction in total NPCs and PCs did not occur. The lack of consistent changes in urinary NPC excretion may have been caused by the consumption of vitamin supplements and varied diets by the study participants. In the MGTT, there were significantly more women using vitamin supplements in the GTE group than the placebo group [27]. Thus, differences in diet and supplement intake could be masking changes in the total NPCs that may have been caused by EGCG consumption. Furthermore, urine samples were collected from the MGTT participants as 24-hour urine samples without dietary restrictions during the collection periods. Therefore, differences in dietary intake among the subjects may have heightened variability in urinary excretion of NPCs and PCs in the present study would be more susceptible to the effects of dietary intake, causing increased variability in the total of the individually measured peaks. Tea catechins also undergo extensive microbial catabolism and gut microflora variability can significantly alter their bioavailability and biological effects [15] [33] [34] [35].

## 5. Conclusion

In conclusion, supplementing 800 mg EGCG/d for 12 months demonstrates slight *in vivo* antioxidant potential in healthy postmenopausal women consuming varied diets. Thus, GTCs such as EGCG could aid slightly in limiting oxidative damage to biomolecules, particularly lipid membranes. This property could protect against oxidative damage that is associated with chronic diseases. However, more long-term intervention studies are needed in subjects that are experiencing greater oxidative stress in order to understand to what extent EGCG functions as an antioxidant *in vivo* in humans [36].

## Figures and Tables

**Table 1.** Catechin and caffeine contents of green tea extract capsules used in the Minnesota Green Tea Trial.

Component	Quantity per capsule (mg)	Dose per day (mg)	Quantity per capsule (%)
Total catechins	328.8 (28.9)	1315.3 (115.4)	80.7
Epigallocatechin (EGC)	26.7 (29.7)	106.8 (118.8)	6.6
Catechin	3.8 (2.1)	15.2 (8.4)	0.9
Epicatechin (EC)	26.8 (5.9)	107.2 (23.4)	6.6
Epigallocatechin gallate (EGCG)	210.7 (11.0)	842.8 (44.1)	51.7
Gallocatechin gallate	8.4 (1.8)	33.6 (7.3)	2.1
Epicatechin gallate (ECG)	50.6 (18.5)	202.4 (74.0)	12.4
Catechin gallate	1.1 (0.5)	4.2 (1.9)	0.3
Gallocatechin	1.3 (1.4)	5.1 (5.6)	0.3
Caffeine	3.9	15.8	1.0

Values are means (SD) from eight batches. Catechin analyses were conducted by Covance Laboratories (Madison, WI). Each capsule’s entire fill weight equals 407.3 mg. Table adapted from ref. Samavat 2015 [27].

**Table 2.** Contents of placebo capsules used in the Minnesota Green Tea Trial.

Component	Quantity per capsule (mg)	Dose per day (mg)	Quantity per capsule (%)
Maltodextrin	204	816	50
Cellulose	202	808	49.5
Magnesium stearate	2	8	0.5

Table adapted from ref. Dostal 2015 [36].

**Table 3.** Urinary excretion of nonpolar lipid peroxidation products measured by HPLC and LC-MS analysis.

Variable	Green Tea Extract (GTE)	Placebo
Total Peak at 30.03 min (100%) (ng hexanal equivalent/mg creatinine)		
0 months	78.02 ± 17.90	94.04 ± 15.36
12 months	50.07 ± 9.45^a,b^	88.66 ± 12.51
Nonanal (90%) (ng/mg creatinine)		
0 months	99.64 ± 22.86	120.10 ± 19.61
12 months	63.94 ± 12.07^a,b^	113.23 ± 15.98
Decatrienal (10%) (ng/mg creatinine)		
0 months	14.92 ± 2.68	16.66 ± 2.83
12 months	10.76 ± 2.19^a,b^	15.12 ± 2.29

Data expressed as mean ± SEM. Total area of nonpolar peak at 30.03 min was determined by HPLC analysis. Urinary excretion of nonanal and decatrienal was determined by LC-MS analysis. ^a^Significantly different from placebo, p < 0.05. ^b^Significantly different from both groups at 0 months, p < 0.005.
